# Chemotherapy significantly improves long-term survival of small lesion node negative metaplastic breast carcinoma in T1c population rather than T1a and T1b

**DOI:** 10.1038/s41598-022-04946-0

**Published:** 2022-01-18

**Authors:** Qitong Chen, Qin Zhou, Hongye He, Yeqing He, Yunchang Yuan, Qiongyan Zou, Wenjun Yi

**Affiliations:** 1 Department of General Surgery, The Second Xiangya Hospital of Central South University, Changsha, 410000 Hunan China; 2 Department of Thoracic Surgery, The Second Xiangya Hospital of Central South University, Changsha, 410000 Hunan China

**Keywords:** Cancer, Diseases, Medical research, Oncology

## Abstract

Metaplastic breast carcinoma (MpBC) is considered a highly aggressive disease, the outcome of chemotherapy on small lesions (T1abcN0M0) MpBC patients remain unclear. We identified 890 female MpBC patients in the Surveillance, Epidemiology, and End Results (SEER) database from 2000 to 2016. After propensity score matching (PSM), 584 patients were matched. Survival probability was compared among T1a, T1b, and T1c patients and between patients with and without chemotherapy using Kaplan–Meier analysis and Cox proportional hazard analysis. Significance was set at two-sided *P* < 0.05. We classified 49, 166, and 675 patients as T1a, T1b, and T1c MpBC, respectively. The chemotherapy group included 404 patients (45.4%). Following PSM, survival analysis indicated that the patients who underwent chemotherapy had higher OS (*P* = 0.0002) and BCSS (*P* = 0.0276) in the T1c substage, but no significant difference was detected in T1a or T1b patients. In this population-based study, small lesion MpBC showed a favorable prognosis. Chemotherapy improved the prognosis of T1c MpBC patients but not T1a and T1b patients to a beneficial extent. Our findings may offer novel insight into a therapeutic strategy for MpBC.

## Introduction

Metaplastic breast carcinoma (MpBC), characterized by mixed epithelial and mesenchymal differentiation, is a rare subtype of primary breast malignancy representing approximately 0.2–1.0%^1,2^. Previous reports suggest that MpBC tends to be aggressive and has an inferior prognosis^3^. Generally, these tumors have multiple features correlated with a poor prognosis similar to triple-negative breast cancer (TNBC), such as larger tumors^4^, poorly differentiated grade, and more hormone receptor and HER2 negativity^2,5^.

Additionally, treatment for MpBC is relatively unelucidated because of the low incidence. Since the current clinical treatment guidelines are based on conventional invasive ductal carcinoma (IDC), more clinical evidence is needed to improve the management strategies for MpBC patients^6^. Chemotherapy is an essential component of breast cancer therapy; however, there is little evidence to support that standard breast cancer chemotherapy regimens utilized for IDC are effective for women with MpBC. Various studies have indicated that patients with T1abcN0M0 breast tumors generally have a favorable prognosis^7^. Nevertheless, there is no exception for MpBC in that outcomes vary among different breast cancer subtypes. The efficacy of chemotherapy for MpBC, especially for small mass lesions (T1a [1–5 mm], T1b [5–10 mm], T1c [10–20 mm] stage) and lymph node-negative, remains unclear.

In the present study, we aimed to explore and identify the survival benefit of chemotherapy in MpBC patients based on data from the Surveillance, Epidemiology, and End Results (SEER) database^8^. Therefore, we performed a retrospective study according to the data of a 890 primary MpBC (T1abcN0M0) population diagnosed without distant organ metastasis between 2000 and 2016. We applied statistical methods such as PSM and Cox analysis models to control the selection bias and balance the disturbance of confounding factors. Our study provides a novel understanding of chemotherapy for small mass lesion MpBC without nodal involvement and distant metastasis and theoretical evidence to solidify the treatment guidelines.

## Materials and methods

### Database and cohort selection

The SEER database registry program sponsored by the National Cancer Institute collects information on all newly diagnosed cancer cases in SEER participating areas in the USA. The demographic, clinicopathological, treatment and outcome information data of MpBC patients were acquired from the SEER database [Incidence- SEER 18 Regs Custom Data (with additional treatment fields), Nov 2018 Sub] via SEER*Stat version 8.3.8 software (https://seer.cancer.gov/seerstat/) in a client server model with permission from the SEER program office.

**Figure 1:**
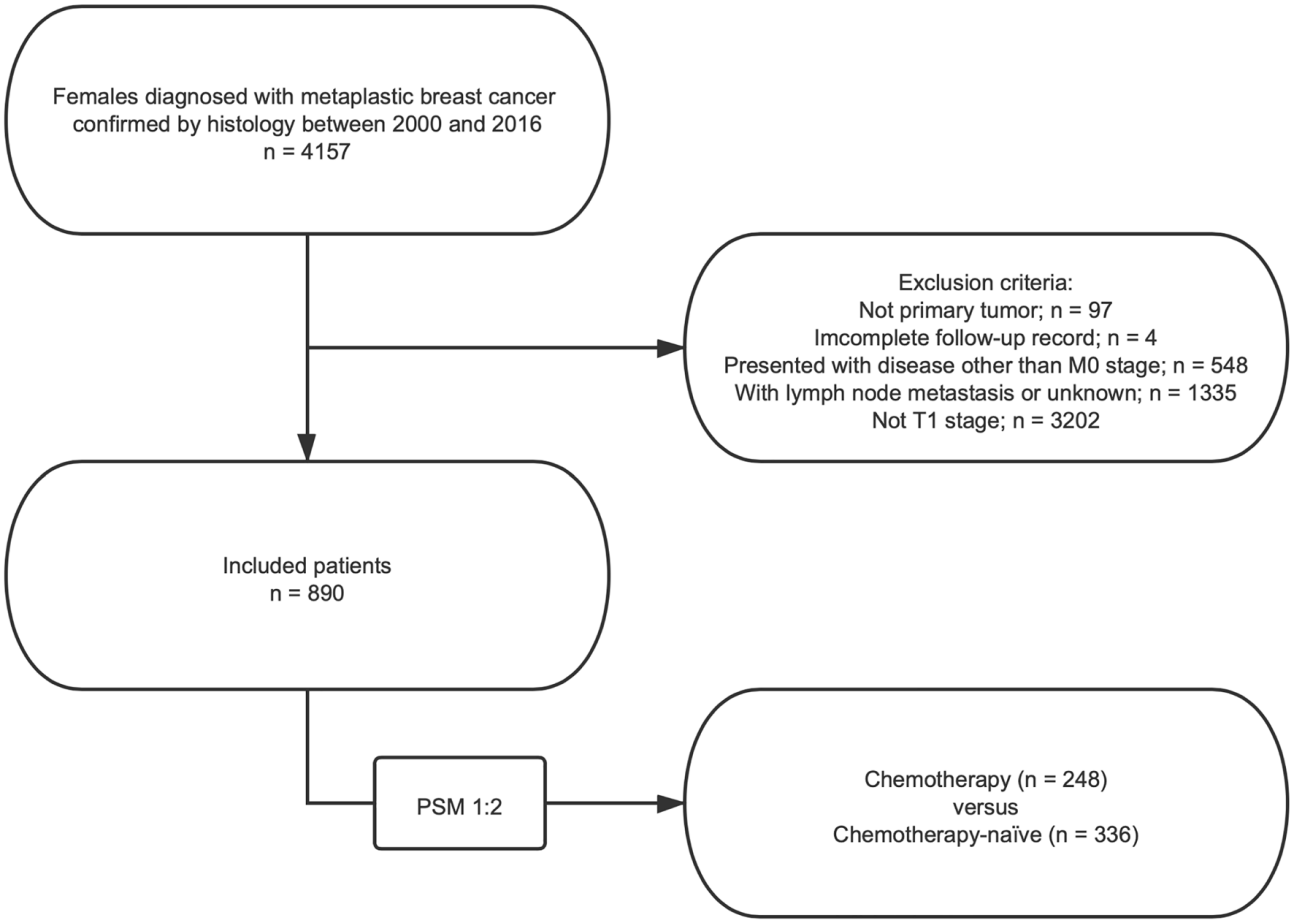
Flow diagram for the study cohort. Abbreviation: PSM, propensity score matching.

Patients diagnosed with pathologically confirmed MpBC from 2000 to 2016 were enrolled in the study. Patients were included if they met the following criteria: (1) female; (2) age at diagnosis over 18 years; (3) breast cancer diagnosis (ICD-0–3 primary site codes: C500-C506, C508, and C509); and (4) histology showing metaplastic carcinoma (ICD-0–3 morphology codes: 8032, 8035, 8052, 8070, 8071, 8072, 8073, 8074, 8075, 8560, 8562, 8570, 8571, 8572, 8573, 8575, 8980, 8981, 8982)^9,10^. MpBC patients who met the following criteria were excluded: (1) not primary tumor when diagnosed with MpBC; (2) had incomplete follow-up data; (3) presented with disease other than AJCC M0 stage disease (M1 or MX); and (4) presented with disease other than AJCC N0 stage disease. Ultimately, a total of 890 female patients with primary MpBC without distant metastasis were chosen. The flow diagram of the patient selection process is presented in Fig. 1.

### Variables

The following demographic, clinicopathologic characteristics and treatment information of MpBC patients before (Supplementary table 1) and after PSM (Table 1) were included: age at diagnosis, marital status, race, median household income, year of diagnosis, grade, breast-adjusted T stage, N stage based on the AJCC-TNM stage system, estrogen receptor (ER) status, progesterone receptor (PR) status, HER2 (human epidermal growth factor receptor 2) status, molecular subtype, surgery of primary site, radiotherapy status, and chemotherapy status. The SEER database started to document HER2 status data from January 2010 so that a section of patients enrolled in the present study had an unavailable HER2 status^11^. Continuous variables, age at diagnosis, were transformed into categorical variables (≤ 45, 46–65, and > 65). Analyses of survival (months), vital status, and cause-specific death classification were performed to evaluate prognostic outcomes.

**Table 1:** Characteristics of female patients diagnosed with primary MpBC in SEER database.

Characteristics	After PSM, n (%)			*P-value*^*a*^
	Overall	Chemotherapy	Chemotherapy-naïve/Unknown	
Sample size	584	248	336	
**Age**				
≤ 45	58 (9.9)	32 (12.9)	26 (7.7)	0.138
46–65	269 (46.1)	119 (48.0)	150 (44.6)	
> 65	257 (44.0)	97 (39.1)	160 (47.6)	
**Marriage**				
Married	331 (56.7)	136 (54.8)	195 (58.0)	0.368
Single	71 (12.2)	37 (14.9)	34 (10.1)	
DSW	145 (24.8)	59 (23.8)	86 (25.6)	
Unknown	37 (6.3)	16 (6.5)	21 (6.2)	
**Race**				
White	468 (80.1)	199 (80.2)	269 (80.1)	0.199
Black	80 (13.7)	36 (14.5)	44 (13.1)	
Other	30 (5.1)	13 (5.2)	17 (5.1)	
Unknown	6 (1.0)	0 (0.0)	6 (1.8)	
**Median household income**				
< $50,000	157 (26.9)	54 (21.8)	103 (30.7)	0.048
$50,000 ~ 70,000	311 (53.3)	144 (58.1)	167 (49.7)	
> $70,000	116 (19.9)	50 (20.2)	66 (19.6)	
**Year**				
2000–2003	91 (15.6)	36 (14.5)	55 (16.4)	0.717
2004–2008	160 (27.4)	65 (26.2)	95 (28.3)	
2009–2012	163 (27.9)	69 (27.8)	94 (28.0)	
2013–2016	170 (29.1)	78 (31.5)	92 (27.4)	
**Grade**				
I–II	178 (30.5)	70 (28.2)	108 (32.1)	0.574
III–IV	319 (54.6)	141 (56.9)	178 (53.0)	
Unknown	87 (14.9)	37 (14.9)	50 (14.9)	
**T stage**				
T1a	26 (4.5)	9 (3.6)	17 (5.1)	0.345
T1b	116 (19.9)	44 (17.7)	72 (21.4)	
T1c	442 (75.7)	195 (78.6)	247 (73.5)	
**ER**				
Positive	104 (17.8)	49 (19.8)	55 (16.4)	0.322
Negative	441 (75.5)	186 (75.0)	255 (75.9)	
Unknown	39 (6.7)	13 (5.2)	26 (7.7)	
**PR**				
Positive	91 (15.6)	43 (17.3)	48 (14.3)	0.364
Negative	452 (77.4)	191 (77.0)	261 (77.7)	
Unknown	41 (7.0)	14 (5.6)	27 (8.0)	
**HER2**				
Positive	11 (1.9)	6 (2.4)	5 (1.5)	0.797
Negative	277 (47.4)	120 (48.4)	157 (46.7)	
Unknown	21 (3.6)	8 (3.2)	13 (3.9)	
Unavailable	275 (47.1)	114 (46.0)	161 (47.9)	
**Molecular Subtype**				
HR + /HER2-	79 (13.5)	38 (15.3)	41 (12.2)	0.578
HER2 enriched	11 (1.9)	6 (2.4)	5 (1.5)	
TNBC	197 (33.7)	82 (33.1)	115 (34.2)	
Unknown	297 (50.9)	122 (49.2)	175 (52.1)	
**Surgery**				
Non-surgery	8 (1.4)	2 (0.8)	6 (1.8)	0.518
Surgery	576 (98.6)	246 (99.2)	330 (98.2)	
**Radiation**				
Radiation	287 (49.1)	128 (51.6)	159 (47.3)	0.346
Non-radiation/Unknown	297 (50.9)	120 (48.4)	177 (52.7)	

^a^*P*-value from Pearson’s chi-square test of independence.

Abbreviations: DSW, divorced/separated/widowed; ER, estrogen receptor; HER2, human epidermal growth receptor 2; HR, hormone receptor; MpBC, Metaplastic breast carcinoma; OS, overall survival; PR progesterone receptor; PSM, propensity score match; TNBC, triple-negative breast cancer.

### Statistical analyses

We conducted descriptive statistics to characterize patient demographics and clinical characteristics. The patient's distribution of clinicopathologic characteristics of chemotherapy and chemotherapy-naïve/unknown groups was assessed using Pearson's χ^2^ test. Overall survival (OS) and breast cancer-specific survival (BCSS) were the primary and secondary endpoints of our study, respectively. OS was defined as that from diagnosis to death due to any cause, and BCSS was determined as the interval from the date of diagnosis to the date of death caused by breast cancer. The Kaplan–Meier curves of OS and BCSS were analyzed by log-rank test. Univariable and multivariable Cox proportional hazard models were applied to evaluate covariates' adjusted effects on OS and BCSS. We compared 5-, 10- and 15-year OS and BCSS rates for T1a, T1b, and T1c tumors across both groups. The efficacy of chemotherapy on OS and BCSS was determined by subgroup analysis, displayed as forest plots. Hazard ratios (HRs), 95% confidence intervals (CIs), and *P*-values were estimated with univariate Cox proportional hazards models of each subgroup. Statistical analyses and data visualization were performed using R 4.0.3 (https://www.r-project.org/). A two-sided *P*-value < 0.05 was regarded as statistically significant.

### Propensity score matching (PSM)

PSM is a reliable statistical method that can control selection bias and balance covariates affecting prognosis in nonrandomized studies^12^. To ensure well-balanced characteristics between the chemotherapy and chemotherapy-naïve/unknown groups, we implemented the "MatchIt" R package 4.1.0^13^ to evaluate propensity scores matched for age, marital status, race, year of diagnosis, grade, T stage, ER, PR, and molecular subtype. The parameter settings of the PSM process were 1:2 pairing, nearest propensity values, and a caliper of 0.10.

## Results

### Baseline characteristics

From 2000 to 2016, 890 patients with T1N0M0 MpBC who had a median age of 63 were included in our study through the SEER database. Age > 65 was reported 44.4% (n = 394). The median follow-up time was 67.5 months. The overall median household income ranged from $50,000 to $70,000. Among 890 patients identified in the original cohort, 49 (5.5%) patients had stage T1a, 166 (18.7%) patients had stage T1b, and 675 (75.8%) patients had stage T1c disease. A total of 18.3% of patients were ER positive, 14.6% of patients were PR positive. Among the available HER2 status and molecular subtype data, 5.3% of patients were HER2 positive and TNBC (273, 66.3%) was the most common. Chemotherapy was administered to 404 patients (45.4%). A total of 49.6% and 98.2% of patients underwent adjuvant radiation therapy and surgery, respectively. Following PSM, a total of 584 patients (chemotherapy n = 248 vs. chemotherapy-naïve/unknown n = 336) were selected for the propensity score-matched cohort. In the matched cohort, 26 (4.5%), 116 (19.9%), and 442 (75.6%) MpBC patients were classified according to stage (T1a, T1b, and T1c, respectively). All variables were balanced adequately between these two groups (Table 1). The baseline characteristics of the patients before and after propensity score matching are summarized in Supplementary table 1 and Table 1.

### Analysis of survival benefits from chemotherapy

**Figure 2:**
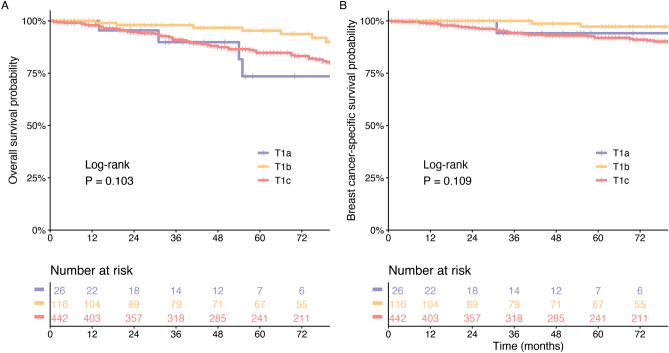
Kaplan–Meier curves comparing the survival of patients with MpBC stratified by T1 stage. (**A**) Overall survival; (**B**) Breast cancer-specific survival. *P*-value was determined by univariate log-rank test.

MpBC patients who underwent chemotherapy (n = 404) had a longer OS (*P* < 0.0001, Supplementary Fig. 1A) than patients who did not. In comparisons of Kaplan–Meier BCSS curves associated with chemotherapy presence or absence, there was a beneficial trend (*P* = 0.0822) identified by log-rank tests (Supplementary Fig. 1C). These results were confirmed by analyzing the PSM cohort (OS: *P* = 0.0001; BCSS: *P* = 0.0350; Supplementary Fig. 1B,D). We compared the Kaplan–Meier curves associated with T1 categories and did not found a significant difference according to the log-rank test for OS (*P* = 0.103, Fig. 2A) and for BCSS (*P* = 0.109, Fig. 2B). Figure 2 demonstrates the survival curves stratified by T1 stage.

**Figure 3:**
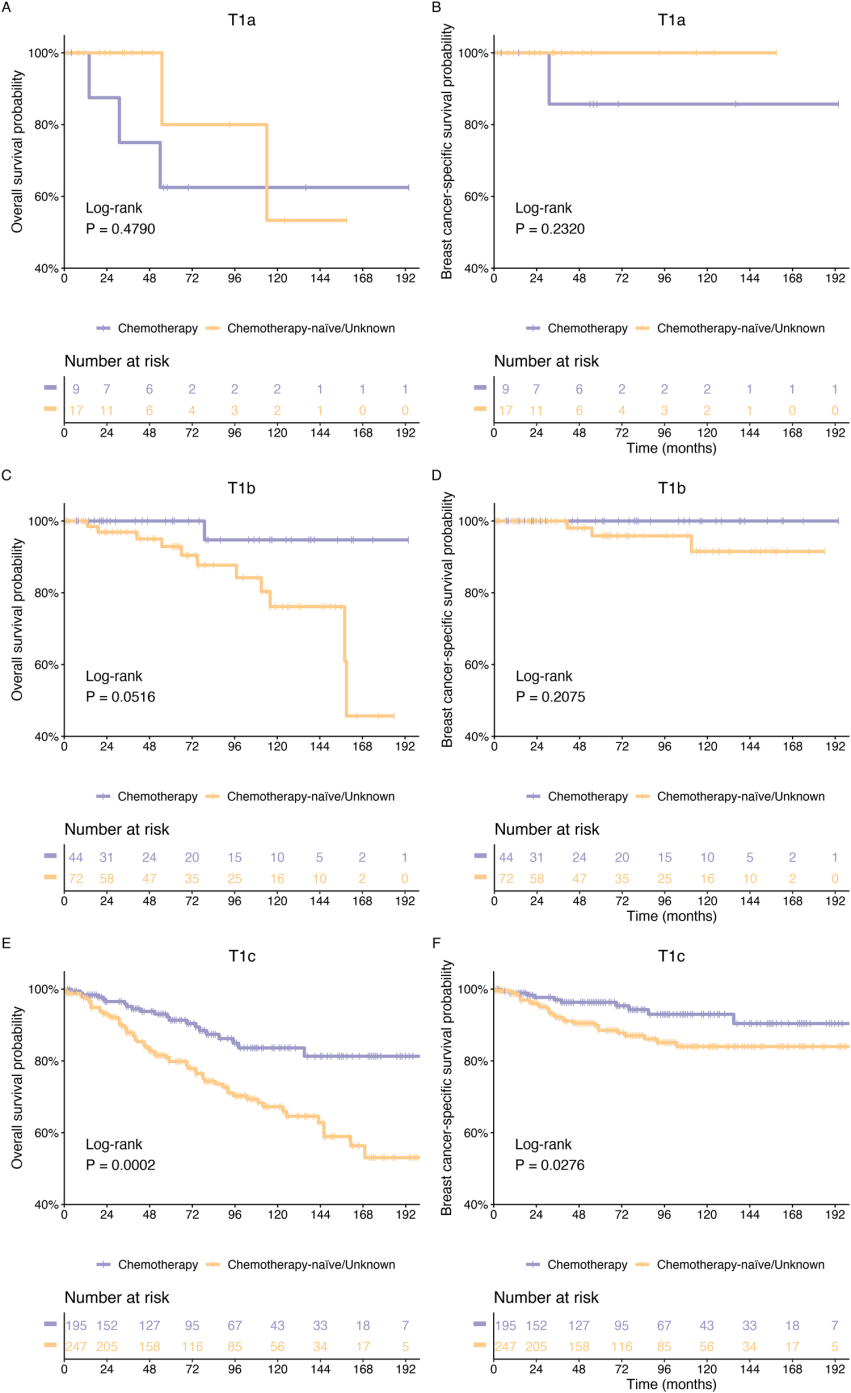
Kaplan–Meier curves comparing survival of patients with MpBC based on chemotherapy and chemotherapy-naïve/unknown (**A**,**B**) Survival analysis of OS and BCSS in the T1a stage subgroup; (**C**,**D**) Survival analysis of OS and BCSS in the T1b stage subgroup; (**E**,**F**) Survival analysis of OS and BCSS in the T1c stage subgroup; *P*-value was determined by univariate log-rank test.

Kaplan–Meier curves for T1a, T1b, and T1c stage patients according to chemotherapy treatment are presented in Fig. 3. In the T1a and T1b patients, no significant difference was found between the chemotherapy and chemotherapy-naïve/unknown groups in either OS (T1a: *P* = 0.479; T1b: *P* = 0.232) or BCSS (T1a: *P* = 0.0516; T1b: *P* = 0.2075) (Fig. 3A–D). In T1c patients, chemotherapy and chemotherapy-naïve/unknown groups had significantly different OS rates (*P* = 0.0002), whereas a beneficial trend was detected in BCSS (*P* = 0.0276). Table 2 shows the 5-, 10-, and 15-year survival rates and 95% CIs for OS and BCSS of MpBC patients stratified by stage at diagnosis.

**Table 2:** T1abc stage MpBC survival outcomes of patients following chemotherapy treatment or not.

Treatment	Overall survival			Breast cancer-specific survival		
	5-year survival (95 CI, %)	10-year survival (95 CI, %)	15-year survival (95 CI, %)	5-year survival (95 CI, %)	10-year survival (95 CI, %)	15-year survival (95 CI, %)
**T1a**						
Chemotherapy	62.500 (0.365–1.000)	62.500 (0.365–1.000)	62.500 (0.365–1.000)	85.714 (0.633–1.000)	85.714 (0.633–1.000)	85.714 (0.633–1.000)
Chemotherapy-naïve/Unknown	80.000 (0.516–1.000)	53.333 (0.214–1.000)	-	100.000 (1.000–1.000)	100.000 (1.000–1.000)	-
**T1b**						
Chemotherapy	100.000 (1.000–1.000)	94.737 (0.852–1.000)	94.737 (0.852–1.000)	100.000 (1.000–1.000)	100.000 (1.000–1.000)	100.000 (1.000–1.000)
Chemotherapy-naïve/Unknown	92.888 (0.863–0.999)	76.138 (0.627–0.924)	45.683 (0.218–0.959)	95.861 (0.904–1.000)	91.503 (0.821–1.000)	91.503 (0.821–1.000)
**T1c**						
Chemotherapy	91.347 (0.869–0.960)	83.623 (0.770–0.909)	81.300 (0.736–0.898)	96.324 (0.935–0.993)	93.000 (0.885–0.978)	90.416 (0.839–0.974)
Chemotherapy-naïve/Unknown	79.842 (0.745–0.855)	67.236 (0.602–0.751)	53.046 (0.426–0.660)	88.547 (0.842–0.931)	83.992 (0.784–0.900)	83.992 (0.784–0.900)

Abbreviations: CI, confidence interval.

### Univariate and multivariate analyses

Univariate Cox analysis revealed that age > 65 years (*P* = 0.001), nonchemotherapy (*P* < 0.001), DSW marital status (*P* = 0.007), III–IV grade (*P* = 0.080), T stage (*P* = 0.087) and negative PR status (*P* = 0.051) might associated with worse outcomes. Multivariable Cox regression analyses showed that age > 65 years (*P* = 0.003), T stage (*P* = 0.027), and chemotherapy (*P* = 0.001) might be associated with OS after adjusting for other prognostic factors. In the univariate Cox regression analysis for BCSS, single (*P* = 0.008) and DSW (*P* = 0.007) marital status and grade III–IV (*P* = 0.034), year of diagnosis 2000–2003 (*P* = 0.092) and chemotherapy-naïve (*P* = 0.039) were associated with worse BCSS. In the multivariate model, single (*P* = 0.015) and DSW (*P* = 0.006) marital status, grade III–IV (*P* = 0.023) and chemotherapy-naïve (*P* = 0.016) predicted worse BCSS. The results of the univariate and multivariate Cox analyses are presented in Tables 3 and 4.

**Table 3:** Univariate and multivariate analysis of overall survival of MpBC patients.

Variables	Univariate analysis			Multivariate analysis		
	Hazard ratio	95% CI	*P*-value	Hazard ratio	95% CI	*P*-value
**Age**						
≤ 45	Ref			Ref		
46–65	2.387	0.734–7.762	0.148	2.205	0.664–7.324	0.197
> 65	6.959	2.180–22.213	0.001	5.940	1.824–19.338	0.003
**Year**						
2000–2003	Ref			–		
2004–2008	0.941	0.568–1.560	0.814			
2009–2012	0.964	0.529–1.758	0.905			
2013–2016	1.557	0.708–3.424	0.271			
**Marriage**						
Married	Ref			Ref		
Single	1.483	0.804–2.734	0.207	1.852	0.995–3.446	0.052
DSW	1.827	1.183–2.821	0.007	1.482	0.949–2.314	0.084
Unknown	0.974	0.417–2.276	0.952	0.953	0.406–2.235	0.912
**Median household income**						
< $50,000	Ref			–		
$50,000 ~ 70,000	1.116	0.699–1.782	0.646			
> $70,000	1.291	0.736–2.264	0.372			
**Grade**						
I–II	Ref			Ref		
III–IV	1.546	0.949–2.521	0.080	1.500	0.915–2.460	0.108
Unknown	1.662	0.913–3.026	0.096	1.591	0.863–2.932	0.137
**T stage**						
T1a	Ref			Ref		
T1b	0.401	0.141–1.141	0.087	0.303	0.105–0.875	0.027
T1c	0.725	0.294–1.787	0.484	0.537	0.214–1.347	0.185
**ER**						
Positive	Ref			–		
Negative	0.943	0.557–1.596	0.827			
Unknown	0.985	0.450–2.157	0.969			
**PR**						
Positive	Ref			Ref		
Negative	1.985	0.998–3.948	0.051	1.653	0.826–3.308	0.156
Unknown	1.751	0.711–4.316	0.223	1.944	0.765–4.940	0.162
**Surgery**						
Non-surgery	Ref			–		
Yes	0.648	0.159–2.631	0.544			
**Radiation**						
Yes	Ref			–		
Nonradiation/unknown	1.124	0.764–1.656	0.552			
**Chemotherapy**						
Yes	Ref			Ref		
Naïve/unknown	2.343	1.493–3.677	< 0.001	2.195	1.393–3.461	0.001

Abbreviations: CI, confidence interval; DSW, divorced/separated/widowed; ER, estrogen receptor; MpBC, metaplastic breast carcinoma; PR progesterone receptor.

**Table 4:** Univariate and multivariate analysis of breast cancer-specific survival of MpBC patients.

Variables	Univariate analysis			Multivariate analysis		
	Hazard ratio	95% CI	*P*-value	Hazard ratio	95% CI	*P*-value
**Age**						
≤ 45	Ref			–		
46–65	0.884	0.252–3.101	0.847			
> 65	2.368	0.714–7.853	0.159			
**Year**						
2000–2003	Ref			Ref		
2004–2008	0.513	0.236–1.114	0.092	0.536	0.246–1.170	0.117
2009–2012	0.599	0.257–1.397	0.235	0.660	0.281–1.551	0.341
2013–2016	0.999	0.355–2.817	0.999	0.994	0.350–2.822	0.991
**Marriage**						
Married	Ref			Ref		
Single	2.963	1.256–6.992	0.013	2.939	1.233–7.009	0.015
DSW	2.750	1.373–5.510	0.004	2.639	1.313–5.306	0.006
Unknown	1.119	0.256–4.897	0.881	1.048	0.239–4.598	0.951
**Median household income**						
< $50,000	Ref			–		
$50,000 ~ 70,000	1.061	0.515–2.188	0.872			
> $70,000	1.162	0.481–2.805	0.738			
**Grade**						
I–II	Ref			Ref		
III–IV	2.970	1.239–7.119	0.015	2.781	1.150–6.725	0.023
Unknown	1.544	0.471–5.062	0.474	1.403	0.424–4.641	0.579
**T stage**						
T1a	Ref			–		
T1b	0.530	0.055–5.099	0.582			
T1c	1.704	0.234–12.424	0.599			
**ER**						
Positive	Ref			–		
Negative	0.996	0.439–2.258	0.993			
Unknown	0.811	0.209–3.147	0.762			
**PR**						
Positive	Ref			–		
Negative	1.424	0.557–3.641	0.461			
Unknown	1.028	0.245–4.311	0.969			
**Surgery**						
Non-surgery	Ref			–		
Yes	0.493	0.068–3.592	0.485			
**Radiation**						
Yes	Ref			–		
Nonradiation/unknown	1.298	0.706–2.385	0.401			
**Chemotherapy**						
Yes	Ref			Ref		
Naïve/unknown	2.063	1.037–4.105	0.039	2.346	1.170–4.705	0.016

Abbreviations: CI, confidence interval; DSW, divorced/separated/widowed; ER, estrogen receptor; MpBC, metaplastic breast carcinoma; PR progesterone receptor.

### Subgroup analysis

**Figure 4:**
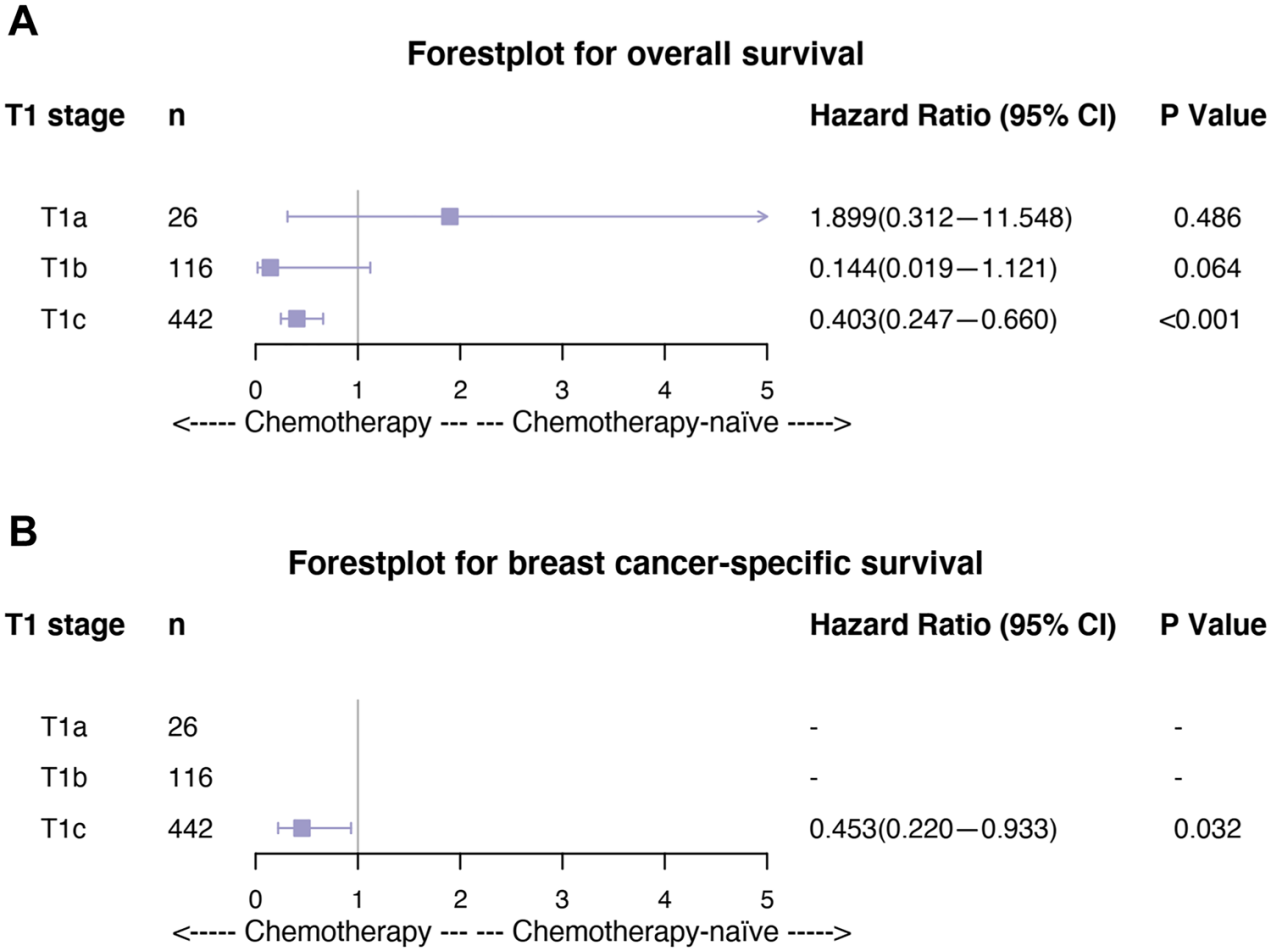
Forest plot of different T1 stage subgroups of MpBC patients. (**A**) Overall survival; (**B**) breast cancer-specific survival. *P-*values are from univariate log-rank tests, and hazard ratios (HRs) and 95% confidence intervals (CIs) are shown. Statistical inference was not available in some subsets due to the small sample size and limited events.

Subgroup analyses to estimate the role of chemotherapy were conducted. The results are shown as forest plots of HR and 95% CI for OS (Fig. 4A) and BCSS (Fig. 4B). The risk of death for OS (HR = 0.403; 95% CI, 0.247–0.660; *P* < 0.001) and for BCSS (HR = 0.453; 95% CI, 0.220–0.933; *P* = 0.032) decreased significantly when chemotherapy was performed in T1cN0 MpBC patients. However, T1a and T1b MpBC patients did not benefit from chemotherapy treatment in terms of either OS or BCSS.

## Discussion

Metaplastic breast carcinoma (MpBC) accounts for less than 1.0% of all breast malignancies^1^. MpBC was officially recognized as a distinct histologic subtype of breast malignancy until 2000 and was then classified into seven subtypes in 2012^1,14^. Studies have shown that the tumor size of MpBC is correlated with distant metastasis and OS^15^. Despite the low nodal involvement, MpBC is considered an aggressive breast cancer subtype due to its worse prognosis. Several studies have reported that MpBC is significantly correlated with worse PFS and OS than TNBC^3,5,16^.

The NCCN clinical practice guidelines^17^ suggest that management of MpBC has largely paralleled that of invasive carcinoma and adopt a comprehensive treatment of surgery, chemotherapy, radiotherapy, endocrine therapy, and targeted therapy based on clinicopathological characteristics and staging of the tumor. MpBC is not sensitive to endocrine therapy and targeted molecular therapy because of its biological features^18^. Evidence on diagnosis and treatment options for MpBC is limited; in particular, the efficacy of adjuvant chemotherapy and neoadjuvant chemotherapy is still controversial.

Most of the literature reports that chemotherapy is less effective in MpBC^19^, and it is more likely to develop drug resistance than nonspecial types of invasive breast cancer^20^. Several studies have reported that chemotherapy was associated with a better outcome, although the effect was limited in early-stage cases^18,21^. Few well-designed research studies have focused on the efficacy of chemotherapy in patients with a small lesion and nonmetastatic status (T1abcN0M0) MpBC. More efforts in this direction are urgently needed. In the present study, we found that chemotherapy was associated with better OS (*P* = 0.0001) and BCSS (*P* = 0.0350) in T1N0M0 MpBC patients (Supplementary Fig. 1). Furthermore, we stratified stage T1 tumors into substages T1a, T1b, and T1c to investigate the role of chemotherapy in small lesion MpBC. The results indicated that while chemotherapy was present, T1c MpBC patients had improved survival (OS, *P* < 0.001, BCSS, *P* = 0.032). However, MpBC patients with T1a and T1b tumors may not obtain similar benefits from chemotherapy. This result suggested that chemotherapy is likely to be inappropriate for T1a and T1b patients, implying that it may be wise to reduce chemotherapy for this substage. In select patients with high-risk features (e.g., young patients with high-grade histology), adjuvant chemotherapy may be considered. To optimize the treatment of patients with T1 stage MpBC, apart from the T stage, other factors of high risks of recurrence should be estimated.

Considering poor response rate of chemotherapy and low HER2 receptor positive rate of MpBC^19,22^, seeking novel therapeutic targets warrants attention. In our study, we found that 66.3% of MpBC were triple-negative subtype. Similar to prior studies^4^, the majority of metaplastic cases were triple negative. In a recent study of 75 metaplastic cases, PD-L1 (Programmed death-ligand 1) overexpression was observed in 46% MpBC cases^23^. It implies the potential benefit of combining checkpoint inhibitors with conventional chemotherapy in MpBC. MpBC harbors somatic mutations in the PI3K, mTOR, and EGFR pathways^24,25^, abnormal activation of the canonical WNT signaling pathway by FAT1 mutations in MpBC was reported^24^, and MpBC is associated with enrichment of EMT pathways as well as angiogenesis gene sets such as prominent expression of vascular endothelial growth factor (VEGF)^26^. A genomic profiling analysis of 192 MpBC samples indicated that tumor-infiltrating lymphocytes were more commonly observed in high mutational burden tumors^27^. It is prompting interest that these signaling path way could be another potential novel treatment strategy.

This study has limitations. This was a retrospective study with the possibility of selection bias, even though we utilized PSM statistical methods to diminish it and make our results more reliable. In addition, some subsets included few events, which may have led to biases and affected the inference. This study's other limitations include the unavailability of detailed chemotherapy regimens for comparing the role of intensive chemotherapy with less intensive chemotherapy, molecular targeted therapy, and recurrence data for calculating DFS/RFS to demonstrate the role of chemotherapy. Optimal treatment strategies for MpBC are being developed based on growing evidence. Further large-scale clinical trials are required to determine appropriate chemotherapy regimens for T1 MpBC patients. Unfortunately, the SEER database does not provide information on biomarkers such as Ki-67, androgen receptor (AR), PD-1, and PD-L1, which are thought to be essential factors affecting prognosis.

In conclusion, chemotherapy improved the prognosis of T1c MpBC patients but not T1a and T1b patients to a beneficial extent. This study could provide evidence-based data that T1a and T1b stage MpBC may not be benefited from chemotherapy. Chemotherapy should be recommended when managing T1c MpBC patients. Further randomized trials are needed to verify these findings.

## Supplementary Information

Supplementary Information.

## Data Availability

The datasets analyzed in the present study can be obtained from the Surveillance, Epidemiology, and End Results (SEER) program online website (https://seer.cancer. gov/). The datasets are also available from the corresponding author upon reasonable request.

## Abbreviations

AJCC: American Joint Committee on Cancer
BCSS: Breast cancer-specific survival
CI: Confidence intervals
ER: Estrogen receptor
HER2: Human epidermal growth factor receptor 2
HR: Hazard ratio
ICD-O-3: International Classification of Diseases for Oncology, third edition
MpBC: Metaplastic breast cancer
OS: Overall survival
PFS: Progression-free survival
PR: Progesterone receptor
SEER: Surveillance, epidemiology, and end results
