# Supplementary material for: Chemotherapy significantly improves long-term survival of small lesion node negative metaplastic breast carcinoma in T1c population rather than T1a and T1b
Source: Sci Rep. 2022 Jan 18;12:871. doi: 10.1038/s41598-022-04946-0 (PMC8766593; doi:10.1038/s41598-022-04946-0)
Supplement: Supplementary file 1 — Supplementary Information. [file 41598_2022_4946_MOESM1_ESM.pdf]

# Title: Chemotherapy Significantly Improves Long-term Survival of Small Lesion Node Negative Metaplastic Breast Carcinoma in T1c population Rather than T1a and T1b

**Authors**
Qitong Chen[1], Qin Zhou[1], Hongye He[1], Yeqing He[1], Yunchang Yuan[2], Qiongyan Zou[1, *], Wenjun Yi[1, *]

Affiliation(s):
1. Department of General Surgery, The Second Xiangya Hospital, Central South University, Changsha, 410000, Hunan, China;
2. Department of Thoracic Surgery, The Second Xiangya Hospital, Central South University, Changsha, 410000, Hunan, China.

Corresponding Authors:

Prof Qiongyan Zou, zqy4311@csu.edu.cn;
Prof Wenjun Yi, yiwenjun@csu.edu.cn.

**Supplementary table 1.** Characteristics of female patients diagnosed with primary MpBC in SEER database

| Characteristics | Before PSM, n (%) | | | |
|---|---|---|---|---|
| | Overall | Chemotherapy | Chemotherapy-naïve/Unknown | *P-value*[a] |
| Sample size | 890 | 404 | 486 | |
| **Age** | | | | |
| ≤45 | 92 (10.3) | 62 (15.3) | 30 (6.2) | <0.001 |
| 46-65 | 404 (45.4) | 239 (59.2) | 165 (34.0) | |
| >65 | 394 (44.3) | 103 (25.5) | 291 (59.9) | |
| **Marriage** | | | | |
| Married | 493 (55.4) | 249 (61.6) | 244 (50.2) | <0.001 |
| Single | 95 (10.7) | 54 (13.4) | 41 (8.4) | |
| DSW | 250 (28.1) | 80 (19.8) | 170 (35.0) | |
| Unknown | 52 (5.8) | 21 (5.2) | 31 (6.4) | |
| **Race** | | | | |
| White | 709 (79.7) | 310 (76.7) | 399 (82.1) | 0.058 |
| Black | 121 (13.6) | 66 (16.3) | 55 (11.3) | |
| Other | 51 (5.7) | 26 (6.4) | 25 (5.1) | |
| Unknown | 9 (1.0) | 2 (0.5) | 7 (1.4) | |
| **Median household income** | | | | |
| <$50000 | 230 (25.8) | 92 (22.8) | 138 (28.4) | 0.159 |
| $50000~70000 | 472 (53.0) | 222 (55.0) | 250 (51.4) | |
| >$70000 | 188 (21.1) | 90 (22.3) | 98 (20.2) | |
| **Year** | | | | |
| 2000-2003 | 151 (17.0) | 53 (13.1) | 98 (20.2) | <0.001 |
| 2004-2008 | 249 (28.0) | 99 (24.5) | 150 (30.9) | |
| 2009-2012 | 235 (26.4) | 113 (28.0) | 122 (25.1) | |
| 2013-2016 | 255 (28.7) | 139 (34.4) | 116 (23.9) | |
| **Grade** | | | | |
| I–II | 275 (30.9) | 74 (18.3) | 201 (41.4) | <0.001 |
| III–IV | 480 (53.9) | 285 (70.5) | 195 (40.1) | |
| Unknown | 135 (15.2) | 45 (11.1) | 90 (18.5) | |
| **T stage** | | | | |
| T1a | 49 (5.5) | 11 (2.7) | 38 (7.8) | <0.001 |
| T1b | 166 (18.7) | 47 (11.6) | 119 (24.5) | |
| T1c | 675 (75.8) | 346 (85.6) | 329 (67.7) | |
| **ER** | | | | |
| Positive | 163 (18.3) | 68 (16.8) | 95 (19.5) | <0.001 |
| Negative | 645 (72.5) | 323 (80.0) | 322 (66.3) | |
| Unknown | 82 (9.2) | 13 (3.2) | 69 (14.2) | |
| **PR** | | | | |
| Positive | 130 (14.6) | 55 (13.6) | 75 (15.4) | <0.001 |

| | | | | |
|---|---|---|---|---|
| Negative | 674 (75.7) | 333 (82.4) | 341 (70.2) | |
| Unknown | 86 (9.7) | 16 (4.0) | 70 (14.4) | |
| **HER2** | | | | |
| Positive | 24 (2.7) | 19 (4.7) | 5 (1.0) | <0.001 |
| Negative | 392 (44.0) | 200 (49.5) | 192 (39.5) | |
| Unknown | 36 (4.0) | 11 (2.7) | 25 (5.1) | |
| Unavailable | 438 (49.2) | 174 (43.1) | 264 (54.3) | |
| **Molecular Subtype** | | | | |
| HR+/HER2- | 115 (12.9) | 53 (13.1) | 62 (12.8) | <0.001 |
| HER2 enriched | 24 (2.7) | 19 (4.7) | 5 (1.0) | |
| TNBC | 273 (30.7) | 145 (35.9) | 128 (26.3) | |
| Unknown | 478 (53.7) | 187 (46.3) | 291 (59.9) | |
| **Surgery** | | | | |
| Non-surgery | 16 (1.8) | 3 (0.7) | 13 (2.7) | 0.057 |
| Surgery | 874 (98.2) | 401 (99.3) | 473 (97.3) | |
| **Radiation** | | | | |
| Radiation | 441 (49.6) | 230 (56.9) | 211 (43.4) | <0.001 |
| Non-radiation/Unknown | 449 (50.4) | 174 (43.1) | 275 (56.6) | |

[a]*P*-value from Pearson's chi-square test of independence.

Abbreviations: DSW, divorced/separated/widowed; ER, estrogen receptor; HER2, human epidermal growth receptor 2; HR, hormone receptor; MpBC, Metaplastic breast carcinoma; OS, overall survival; PR progesterone receptor; PSM, propensity score match; TNBC, triple-negative breast cancer.

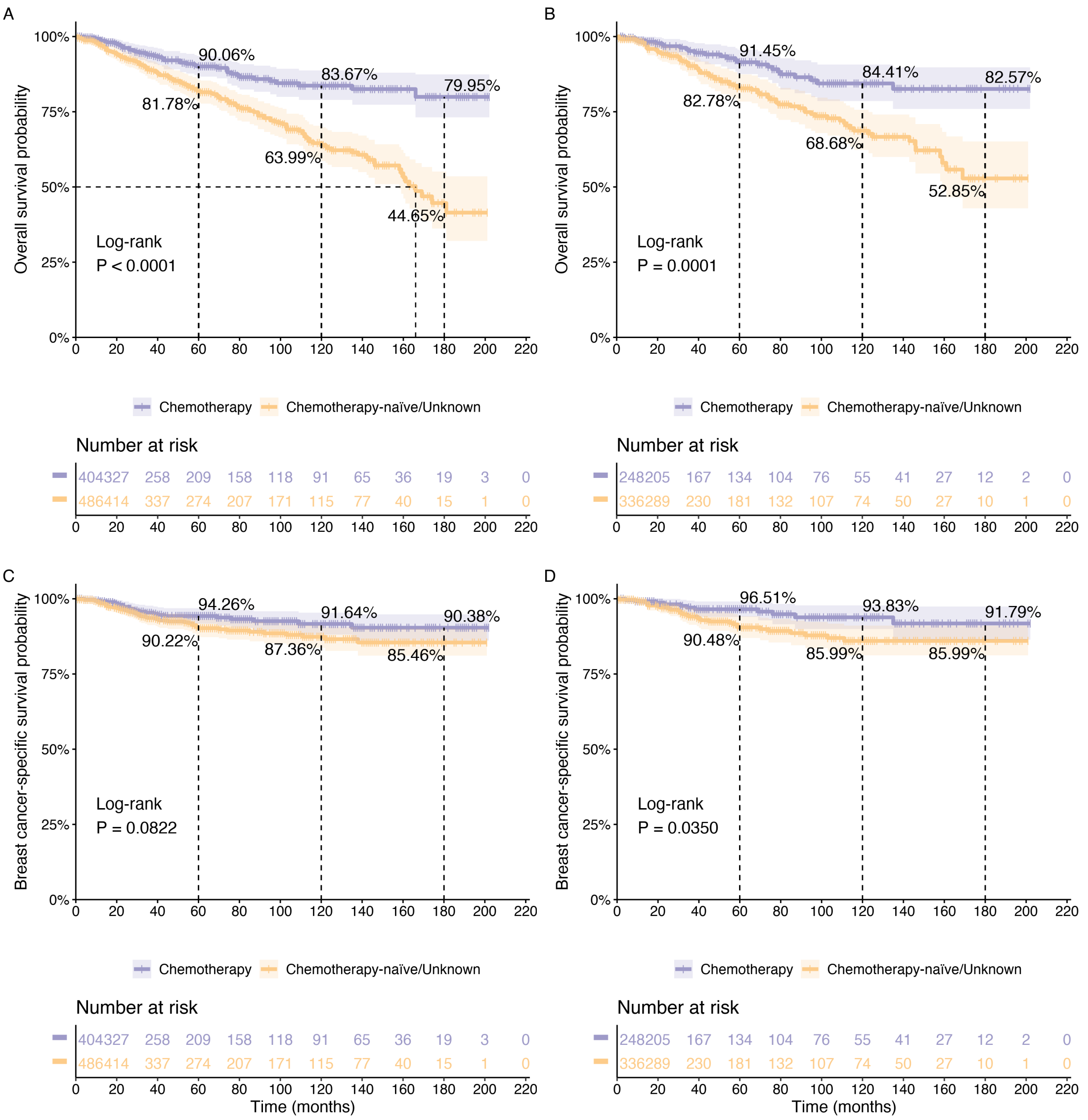


Supplementary Figure 1 Kaplan–Meier curves comparing survival of patients with MpBC based on chemotherapy and chemotherapy-naïve/unknown for overall survival (OS) and breast cancer-specific survival (BCSS). (**A**) (**B**) Survival analysis of OS before and after propensity score matching (PSM); (**C**) (**D**) Survival analysis of BCSS in the T1b stage subgroup
